# How to understand real net ultrafiltration and its association with low blood pressure in critically ill patients with renal replacement therapy

**DOI:** 10.1186/s13054-018-2298-0

**Published:** 2019-01-23

**Authors:** Wei Li, Jinsong Zhang, Xufeng Chen

**Affiliations:** Department of Emergency Medicine, The First Affiliated Hospital of Nanjing Medical University, People’s Hospital of Jiangsu Province, Nanjing, 210029 China

## Abstract

The concept of net ultrafiltration (UF^NET^) mentioned in the paper by Murugan et al. in a recent issue of *Critical Care* does not equate to the real UF^NET^ in patients with renal replacement therapy initiation. Furthermore, the baseline blood pressure among the groups had a statistically significant difference. Both of these two factors may affect the final results. Thus, we should be cautious interpreting the conclusions.

In a recent issue of *Critical Care*, Murugan and colleagues drew the conclusion that, among critically ill patients with ≥ 5% fluid overload, the patients with net ultrafiltration (UF^NET^) exceeding 25 ml/kg/day compared with those below 20 ml/kg/day had a lower 1-year risk-adjusted mortality [1]. The definition of UF^NET^ in their paper was calculated as the difference between the volume of ultrafiltration and substitution fluids. Furthermore, for patients receiving continuous venovenous hemodiafiltration and slow continuous ultrafiltration, UF^NET^ corresponded to the volume removed. However, for patients on renal replacement therapy, spontaneous urine output (UO) and intravenous fluid via the peripheral vein (Fiv) should also be taken into account for the estimation of total UF^NET^. Thus, the total net ultrafiltration should be adjusted by UF^NET^ + UO – Fiv. We believe that this adjustment achieves a more accurate parameter to reflect the hemodynamic status of patients in clinical practice.

In addition, as shown in their study, the imbalance was observed for the mean arterial pressure among three different levels of UF^NET^ intensity in all the patients receiving renal replacement therapy and the subset of patients receiving continuous renal replacement therapy (*P* < 0.001). It is well recognized that low blood pressure is a common complication during renal replacement therapy and is strongly associated with illness severity [2]. Low blood pressure will trigger an adjustment for total ultrafiltration volume. Thus, blood pressure appears to be a confounding factor to the results. Although adjusted by statistical models, it is better to re-analyze the association between UF^NET^ intensity and mortality by categorized blood pressure levels to provide stronger evidence.

## Abbreviations

Fiv: Fluid infusion by peripheral vein
UF^NET^: Net ultrafiltration
UO: Urine output
