# Supplementary material for: How to understand real net ultrafiltration and its association with low blood pressure in critically ill patients with renal replacement therapy
Source: Crit Care. 2019 Jan 23;23:20. doi: 10.1186/s13054-018-2298-0 (PMC6343245; doi:10.1186/s13054-018-2298-0)
Supplement: Related texts

# Reply: Authors’ response

**Authors:** Raghavan Murugan, Rinaldo Bellomo, Paul M. Palevsky, John A. Kellum

We would like to thank Dr. Li and colleagues for their thoughtful letter regarding our article [1]. They propose that we should account for urine output as well as the intravenous fluids administered in estimating the total UF^NET^ volume. However, we would like to clarify that the purpose of our study was to examine whether there was an independent association between the process of care variable, UF^NET^ intensity, and risk-adjusted 1-year mortality. Thus, we specifically did not include the urine output and the intravenous fluids in the calculation of UF^NET^ (exposure variable) as it would confound the assessment of the relative contribution of UF^NET^ intensity on the outcome.

In our study, all intravenous fluids administered, as well as fluid losses including the urine output, were part of the input and output equation to calculate the severity of fluid overload before initiation of renal replacement therapy as well as the cumulative fluid balance during renal replacement therapy (as outlined in Additional file 1: methods S2 [1]). The severity of fluid overload before initiation of renal replacement therapy as well as the cumulative fluid balance after initiation of renal replacement therapy were adjusted in all the multivariable regression models (Tables 3, 4, and 5 in [1]).

Nevertheless, we would like to acknowledge that in clinical practice there are variety of factors that are likely to influence the clinical decision to determine the target UF^NET^ (e.g., starting fluid balance, ongoing fluid input/output, patient tolerance of fluid removal, severity of illness and organ edema, etc.) and further research is required to determine the relative contribution of these variables on UF^NET^ intensity and outcomes.

We completely agree with Dr. Li and colleagues that even though we adjusted for mean arterial pressure as well as the vasopressor dose in the models, we cannot exclude the possibility of residual confounding by hemodynamics on UF^NET^ intensity and outcomes. Although we could certainly perform a stratified analysis by mean arterial pressure, it would be difficult to fit models that account for continuous variation in blood pressure throughout the duration of renal replacement therapy and disentangle its association with UF^NET^ intensity and the outcome.
